# No difference in the competitive ability of introduced and native *Trifolium* provenances when grown with soil biota from their introduced and native ranges

**DOI:** 10.1093/aobpla/plw016

**Published:** 2016-03-11

**Authors:** Natasha Shelby, Philip E. Hulme, Wim H. van der Putten, Kevin J. McGinn, Carolin Weser, Richard P. Duncan

**Affiliations:** 1 Bio-Protection Research Centre, Lincoln University, PO Box 85084, Lincoln 7647, New Zealand; 2 Department of Terrestrial Ecology, Netherlands Institute of Ecology (NIOO-KNAW), Droevendaalsesteeg 10, 6708 PB Wageningen, The Netherlands; 3 Laboratory of Nematology, Wageningen University, PO Box 8123, 6700 ES Wageningen, The Netherlands; 4 Institute for Applied Ecology, University of Canberra, Canberra, ACT 2601, Australia

**Keywords:** Alien, competition, enemy-release, exotic, invasive, rhizosphere microbiota, soil biota, weed

## Abstract

The evolution of increased competitive ability (EICA) hypothesis could explain why some introduced plant species perform better outside their native ranges. The EICA hypothesis proposes that introduced plants escape specialist pathogens or herbivores leading to selection for resources to be reallocated away from defence and towards greater competitive ability. We tested the hypothesis that escape from soil-borne enemies has led to increased competitive ability in three non-agricultural *Trifolium* (Fabaceae) species native to Europe that were introduced to New Zealand in the 19th century. *Trifolium* performance is intimately tied to rhizosphere biota. Thus, we grew plants from one introduced (New Zealand) and two native (Spain and the UK) provenances for each of three species in pots inoculated with soil microbiota collected from the rhizosphere beneath conspecifics in the introduced and native ranges. Plants were grown singly and in competition with conspecifics from a different provenance in order to compare competitive ability in the presence of different microbial communities. In contrast to the predictions of the EICA hypothesis, we found no difference in the competitive ability of introduced and native provenances when grown with soil microbiota from either the native or introduced range. Although plants from introduced provenances of two species grew more slowly than native provenances in native-range soils, as predicted by the EICA hypothesis, plants from the introduced provenance were no less competitive than native conspecifics. Overall, the growth rate of plants grown singly was a poor predictor of their competitive ability, highlighting the importance of directly quantifying plant performance in competitive scenarios, rather than relying on surrogate measures such as growth rate.

## Abstract (precis)

This biogeographic study tested the evolution of increased competitive ability (EICA) hypothesis—a compelling explanation for why plants become invasive. We measured growth rates and the competitive ability of three *Trifolium* species sourced from their native (Spain and the UK) and New Zealand-naturalised ranges when grown with rhizosphere microbiota from each range. Although EICA was not supported (naturalised plants were not more competitive) the differences in plant competitive ability when grown with different rhizosphere microbial communities illustrate that soil microbiota affect plant growth and competition. The work illustrates an important finding: growth of singly-grown plants doesn't always predict competitive ability.

## Introduction

One of the most widely examined hypotheses for the success of non-native invasive plants is the evolution of increased competitive ability (EICA) (Blossey and Nötzold 1995; Keane and Crawley 2002; Vestergård *et al.* 2015). The fundamental assumption underpinning EICA is that introduced plants benefit from escaping specialist enemies in their native range (Liu and Stiling 2006). Enemy escape can then select for a shift in energetic investment away from costly defence traits and towards growth (Doorduin and Vrieling 2011), which may lead to greater competitive ability in introduced populations (Blossey and Nötzold 1995). A large body of evidence supports rapid contemporary adaptation of introduced plants to novel environments (Prentis *et al.* 2008); however, few tests find support for the full set of EICA predictions (Bossdorf *et al.* 2005). For example, some introduced plants grow larger than conspecifics in the native range without an apparent loss of defences (Alba *et al.* 2011), while other introduced plants are both larger and better defended (Ridenour *et al.* 2008; Abhilasha and Joshi 2009; Caño *et al.* 2009).

One explanation for the equivocal findings of EICA is that there is no standard metric with which to quantify differences in plant performance. The EICA hypothesis states that introduced plants increase their competitive ability, yet most studies do not measure competition directly, instead using surrogate measures such as growth rate or size to infer competitive ability (Blair and Wolfe 2004; Joshi and Vrieling 2005; Meyer *et al.* 2005; Stastny *et al.* 2005; Franks *et al.* 2008; van Kleunen and Fischer 2008; Huang *et al.* 2010). A recent meta-analysis revealed that of 58 EICA studies, only 10 measured competitive ability directly, and in all cases, the competitive ability of native and introduced provenances was assessed relative to heterospecifics (Felker-Quinn *et al.* 2013). Using a heterospecific as a ‘phytometer’ to measure competitive ability could confound competitive effects with other species-specific interactions (Maron *et al.* 2004), such as allelopathy (Ridenour *et al.* 2008; Qin *et al.* 2013), root architecture (Rubio 2001) and differences in how species cultivate soil microbiota or affect nutrient dynamics (Wilson and Tilman 1993; van der Putten *et al.* 2007). Contemporary tests demonstrate the value of assessing competitive ability in a standardized way and the difficulties associated with selecting an arbitrary heterospecific against which to assess differences between native and introduced provenances (Bossdorf *et al.* 2004; Beaton *et al.* 2011; Liao *et al.* 2013).

Another explanation for the inconsistent results among EICA studies is that experimental designs have not incorporated the soil microbial communities from which plants derive a rhizosphere community in their native and introduced ranges. Soil communities differ between locations (Pringle *et al.* 2009; Litchman 2010; Tedersoo *et al.* 2014) and play determining roles in plant community composition (Coats and Rumpho 2014), plant competition (van der Putten and Peters 1997) and invasions (Callaway *et al.* 2011). For example, antagonistic microbes can limit plant growth, whereas growth-promoting soil endophytes, such as mycorrhizal fungi, can provide a competitive advantage by exponentially increasing root surface area and therefore the acquisition of both water and nutrients (Sabais *et al.* 2012). Other root endophytes induce systemic resistance, making plants better able to combat subsequent enemies and environmental stress (Pieterse *et al.* 2014). Because interactions between plants and soil biota can alter competitive outcomes and influence community composition, escape from soil-borne enemies could be an important factor in the success of introduced plants (Diez *et al.* 2010; Reinhart *et al.* 2010; Callaway *et al.* 2011). However, how the performance of native and introduced conspecifics differs when they are exposed to the microbial communities of each range has only been partially tested (Felker-Quinn *et al.* 2013). For example, the performance of native and introduced *Lygodium microphyllum* (Lygodiaceae) (Volin *et al.* 2010) and *Pinus contorta* (Pinaceae) (Gundale *et al.* 2014) have been shown to differ in soils from the native and introduced ranges, but it is not clear if their competitive abilities have been altered because neither species was grown under competitive conditions.

In this study, we test the EICA hypothesis by comparing the growth rate and competitive ability of introduced and native provenances of the same species when grown with soil microbiota from the introduced and native ranges. We used three species of non-agricultural *Trifolium* (Fabaceae) native to Europe that were introduced to New Zealand in the 1800s and have naturalized widely, including in disturbed, ruderal locales and pastures where competition with grasses, forbs and agricultural congeners is common (Boswell *et al.* 2003; Maxwell 2013). We performed intraspecific competition experiments to test two predictions of the EICA hypothesis: (i) plants from introduced provenances outcompete conspecifics from native provenances when grown with soil microbiota from the introduced range (because introduced plants have evolved greater competitive ability in response to a lack of specialist soil-borne antagonists in the introduced range) and (ii) plants from native provenances outcompete introduced provenances when grown with soil microbiota from the native range (because introduced provenances have lost defences against specialist soil-borne antagonists that are absent in the introduced range).

## Methods

We selected three species of ‘true clovers’, *Trifolium arvense*, *T. campestre* and *T. striatum*, that are native to Europe and widely naturalized in New Zealand. We restricted our study to non-agricultural species so that any differences between native and introduced provenances were not the result of selective agronomic breeding. These species have traits common among plants that adapt rapidly to new conditions: they are annuals that spread by seed, they are predominantly out-crossers and they have been successful in a wide range of habitats following their introduction to many regions worldwide (Boswell *et al.* 2003; Atwood and Meyerson 2011). All three species naturalized in New Zealand before 1876 and have had more than 130 years to adapt to local conditions (Willis *et al.* 2000; Whitney and Gabler 2008) **[see Supporting Information]**.

### Experimental design

#### Rhizosphere soil collection

Glasshouse pots were inoculated with soil containing rhizosphere microbiota that was cultivated *in situ* by conspecific plants of each of the three *Trifolium* species in each range. In the introduced range, soil was collected at five sites for each species from Banks Peninsula, Canterbury, New Zealand. This region comprises a variety of habitats broadly representative of where these species have naturalized on the South Island of New Zealand (Boswell *et al.* 2003). In the native range, we collected soil from five sites for each species in each of two regions: the southern UK and northern Spain. Ideally tests investigating adaptation in introduced plants compare populations and soils from the introduced range with populations and soils from the region of the native range from which the introduced plants originated (Gundale *et al.* 2014). The origin of the founding populations for these accidentally introduced clovers is unknown, but many of New Zealand's agricultural clovers were imported from the UK (Gravuer 2004), making it a likely source location and an appropriate native-range comparison. We also included seed provenances and soils from northern Spain, as the three species are common in this region and the latitude closely matches our sampling locations in New Zealand, which may minimize performance differences associated with latitudinal clines (Colautti *et al.* 2009).

The five soil collection sites in each country were located between 1 and 221 km apart, to encompass a range of soils, rhizosphere microbial communities and land-use types. At all sites, the species of interest co-occurred with congeners, particularly the agricultural species *T. repens*. At each site, we collected ∼10 mL of rhizosphere soil from directly beneath each of 10 plants located at least 1 m apart. Equipment was sterilized between sites to keep replicates independent. Soil from each site was air-dried (Reinhart *et al.* 2003), bulked and sieved to 4 mm. We also removed all visible macrobiota and roots before storing the soils in sealed bags in cool storage rooms (16–22 °C).

#### Seed collection

We sourced seed of each species from one site in the introduced range (New Zealand) and two in the native range (Spain and the UK) **[see Supporting Information]**. Seed was hand-collected from a minimum of 12 plants, homogenized, cleaned and tested for viability prior to the experiments. For *T. arvense* in the UK, seed collected from wild populations was sourced from Herbiseed, a UK germplasm centre, because plants in the field were not setting seed when we collected soil. Although seed from any one population will not capture the genetic diversity in a given range, in this study, species is the intended level of replication. Each *Trifolium* species presumably has its own suite of rhizosphere antagonists and mutualists, and thus, each species forms an independent unit for comparing the performance of plants from native and introduced provenances. In addition, if the EICA hypothesis holds, we expect differences in growth rates and competitive ability between native and introduced provenances to be greater than the differences among populations within each range (Leger and Rice 2003; Buschmann *et al.* 2005; Erfmeier and Bruelheide 2005; Blumenthal and Hufbauer 2007). Seeds were sterilized in a 10 % solution of bleach for 2 min, rinsed thoroughly in deionized water and scarified gently with a scalpel to break the hard seed coat. Seeds were germinated on sterile glass beads under species-specific temperature and day-length requirements in a germination cabinet **[see Supporting Information]**.

#### Glasshouse experiments

To compare the performance of plants from native and introduced provenances in the presence of soil microbiota from each range, we conducted two glasshouse experiments. Experiments were run separately in each range to comply with quarantine regulations and to avoid the potentially confounding effects of different transit and storage conditions. The test with introduced-range soil was carried out at Lincoln University in Canterbury, New Zealand, in Southern Hemisphere summer 2013. This experiment tested the prediction that growth rates and competitive ability would be greater among plants from introduced provenances compared with native conspecifics when grown with soil microbiota from the introduced range as a result of introduced provenances having escaped specialist enemies and diverted resources from defence towards competitive ability. The experiment with soils from the native range was conducted at The Netherlands Institute of Ecology in Wageningen, The Netherlands, in Northern Hemisphere summer 2013. This experiment tested the hypothesis that introduced provenances would grow more slowly and be less competitive than native provenances when exposed to native-range soil microbiota as a result of introduced provenances having shifted resources away from defence against specialist enemies present in native-range soils.

We grew plants from each of the native and introduced provenances alone in single-plant pots and in competition with each other in paired-plant pots. In the single-plant pots, a plant from each provenance was grown singly with an inoculum of rhizosphere soil from one of the five soil collection sites replicated twice to give 90 single-plant pots in New Zealand soil (3 species × 3 provenances × 5 soil sites × 2 replicates) and 60 single-plant pots in each native-range soil (3 species × 2 provenances × 5 soil sites × 2 replicates). In the paired-plant pots, a plant from the introduced provenance was grown in competition with a plant from one of the native provenances (either UK or Spain) with an inoculum of rhizosphere soil from one of the five soil collection sites replicated twice, giving 60 paired-pots in each soil (3 species × 2 native provenances × 5 soil sites × 2 replicates).

The sandy background soils that formed the bulk of each pot were sterilized by two successive rounds of autoclaving (20 min at 121 °C) in New Zealand and by γ irradiation (>25 kGy) in The Netherlands. No fertilizers or soil amendments were used in either glasshouse, as the sterilized background soil provided sufficient nutrients. A 10 % (v/v) inoculum of unsterilized rhizosphere soil was mixed into the background soil in each pot to provide the soil microbiota without strongly influencing other properties of the soil, such as pH, nutrients and organic matter (Maron *et al.* 2004; van der Putten *et al.* 2007). Seedlings were transplanted into the pots soon after they had their first true leaves, and seedlings that died within the first week were replaced. Further mortality occurred within the next 2 weeks but dead seedlings were not replaced so that, at the time of harvest, there were 187 plants from the single-plant pot treatment (60 in Spanish soil, 54 in the UK soil and 73 in New Zealand soil) and 98 plants from the paired-plant pot treatment (30 in Spanish soil, 26 in UK soil and 42 in New Zealand soil). Seedling mortality was low (11 %), occurred early and was not attributable to competition effects.

Pots were assigned to a random location in the glasshouses and moved every 2 weeks. Single-plant pots and paired-plant pots were watered to a species-standardized weight on a weekly or twice-weekly basis as needed. Plants of the same species were harvested on the same day after ∼3 months when plants began forming flower buds, indicating an energetic switch from growth to reproduction, and it was clear that plants were nearing pot capacity. Roots were washed gently and colonization by the nitrogen-fixing symbiont was scored on a 0–3 scale following a modified protocol from Corbin *et al.* (1977) that takes into account the quantity, size, location and colour of nodules **[see Supporting Information]**. Roots and shoots were separated and oven-dried at 65 °C. Growth rate (g day^−1^) was measured as dry-weight biomass/number of glasshouse growing days to standardize comparisons among species.

### Statistical analyses

We first compared the growth rates of singly grown plants using separate linear mixed-effects models for each species, comparing growth rate (log-transformed to meet assumptions of normality and constant variance) in soils from each range (New Zealand, Spain and the UK). We accounted for potential non-independence due to site-specific effects by including the site from which soil was collected as a random effect in the models. Because *Trifolium* growth can depend on the degree of association with its nitrogen-fixing symbiont, and differences in nodulation **[see Supporting Information]** rather than shifts in resource allocation could explain differences in growth rates, we included nodulation score as a fixed effect in our model. Doing this provides an estimate of growth rate having accounted statistically for the effect of nodulation on growth. To test for a significant difference in growth rate among plants from different provenances grown in the same soil, we ran an analysis of variance on the difference between the model that included seed provenance as a fixed effect and the one with seed provenance removed.

To compare the competitive ability of plants from native and introduced seed provenances grown in soil from each range, we computed a relative competition intensity (RCI) index for each native and introduced provenance in each soil. Relative competition intensity is a standard competition index (Weigelt and Jolliffe 2003), calculated as:

$$\text{RC}{\text{I}}_{A(B)}=\frac{\text{G}{\text{R}}_{A}-\text{G}{\text{R}}_{A(B)}\;}{\text{G}{\text{R}}_{A}}$$

where GR_A_ is the growth rate of a plant from provenance A when grown alone and GR_A(B)_ is the growth rate of a plant from provenance A when grown in competition with a plant from provenance B. An RCI_A(B)_ value of 0 indicates there was no competitive effect (i.e. growth rates of provenance A were the same for plants grown singly and in competition with provenance B). Increasing values of RCI_A(B)_ (up to a maximum of 1) indicate increasingly greater competitive strength of provenance B. An RCI_A(B)_ value of <0 would indicate that provenance A grew better with provenance B than singly. Relative competition intensity and similar measures of competition intensity have been widely used in studies of plant competition and allow us to compare our results with the few EICA tests that have included a competition index (Vilà and Weiner 2004; Liao *et al.* 2013; Oduor *et al.* 2013; Qin *et al.* 2013).

For each species, we calculated RCI values by first fitting a linear mixed-effects model to the (log-transformed) growth rates of plants from single-plant and paired-plant treatments in each soil type (New Zealand, Spain and the UK), including the site from which soil was collected as a random effect. We fitted this model without an intercept and with a variable that coded for the seed provenance (for single-plant pots) or seed-provenance combination (for paired-plant pots) as a fixed effect. As with the growth-rate model, we included as a fixed effect the plant's nodulation score to remove its effect. We extracted from this model the mean growth rate and associated uncertainty for each seed provenance and seed-provenance combination having accounted for site effects. We used these mean growth rates and their uncertainties to calculate the RCI indices **[see Supporting Information]** having accounted for any growth differences attributable to site effects and variation in degree of nodulation by the nitrogen-fixing root symbiont.

To allow the uncertainties associated with the estimates of mean growth rate to propagate into the RCI index, we used a simulation approach, extracting the variance–covariance matrix for the fixed effects from the fitted models (Gelman and Su 2014). These variance–covariance matrices provide estimates of the mean growth rate of single and paired plants, along with their variances and co-variances. We then drew 100 000 random values from the normal distributions defined by these variance–covariance matrices to obtain a distribution of estimates of mean growth rates, and used these values to calculate 100 000 values for each RCI index, from which we obtained the means and 95 % confidence intervals. For each species in each soil type (New Zealand, Spain and the UK), we calculated two RCI indices for each native-introduced provenance pair. In New Zealand soil, for example, we calculated RCI_NZ(SP)_, which measures the competition intensity experienced by the introduced (New Zealand) provenance when grown with the native (Spanish) provenance, and we calculated RCI_SP(NZ)_, which measures the competition intensity experienced by the Spanish provenance when grown with the New Zealand provenance.

To compare the competitive ability of native and introduced provenances of each species in each soil, we subtracted the RCI index of the native provenance from the RCI index of the introduced provenance for each of the 100 000 simulated values:

$$\text{RC}{\text{I}}_{\mathrm{NZ}(\mathrm{SP})}-\text{RC}{\text{I}}_{\mathrm{SP}(\mathrm{NZ})}$$

The resulting means and 95 % confidence intervals provide the difference in competitive ability between native and introduced provenances in the same soil, and the associated uncertainty. A value of zero would indicate no difference in competitive ability between seed provenances; values greater than zero indicate the native provenance was more competitive, and negative values indicate the introduced provenance was more competitive. We assessed the significance of these differences by whether the 95 % confidence intervals overlapped zero.

To test whether differences in growth rate translated to differences in competitive ability, we tested for a correlation between the growth-rate differences and the RCI value differences between native and introduced provenances across all species and soils. All statistical analyses were performed using R ver. 3.0.2 (R Development Core Team 2013) and model scripts are provided in **Supporting Information**. Linear mixed-effects models were fitted using the lmer function, which uses restricted maximum likelihood, in the R package ‘arm’ ver. 1.6.10 (Gelman and Su 2014).

## Results

### Growth in the absence of competition

**Figure 1.**
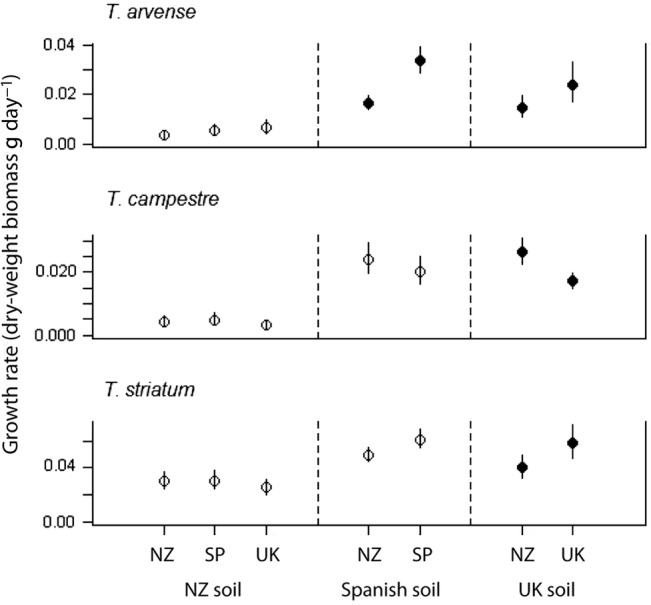
Model-adjusted growth rates of plants from the introduced (New Zealand, NZ) and native (Spanish, SP, and the United Kingdom, UK) seed provenances of three *Trifolium* species grown singly in pots inoculated with rhizosphere soil cultured by conspecifics in New Zealand, Spain and the UK. Error bars are 95 % confidence intervals. Filled circles represent inter-provenance differences that are statistically significant (*P* < 0.05).

When grown in soils from the introduced range, there was no clear difference in the growth rates of native and introduced provenances for any of the three *Trifolium* species (Fig. 1). In native-range soils from Spain and the UK, however, plants from New Zealand provenances of *T. arvense* and *T. striatum* on average grew more slowly than plants from each of the native provenances (Fig. 1). For these two species, the differences in growth rate between provenances were often substantial. The New Zealand provenance of *T. arvense* grew about half as fast on average as the native provenances in both Spanish soil (*F*_1,20_ = 76.34; *P* < 0.001) and in UK soil (*F*_1,15_ = 6.50; *P* = 0.03), while the New Zealand provenance of *T. striatum* grew at about two-thirds the rate of natives in UK soil (*F*_1,19_ = 9.77; *P* = 0.04). The New Zealand provenance of *T. striatum* tended to grow more slowly than Spanish plants in Spanish soil, but this difference was not significant (*F*_1,20_ = 14.39; *P* < 0.93). In contrast, plants of *T. campestre* showed the opposite pattern: plants from the UK provenance had growth rates that were about two-thirds the rate of New Zealand plants grown in UK soil (*F*_1,20_ = 19.44; *P* < 0.001). Native *T. campestre* from Spain also tended to grow more slowly than New Zealand plants in Spanish soil, but this difference was not significant (*F*_1,20_ = 3.08; *P* = 0.90).

### Competitive ability

**Figure 2.**
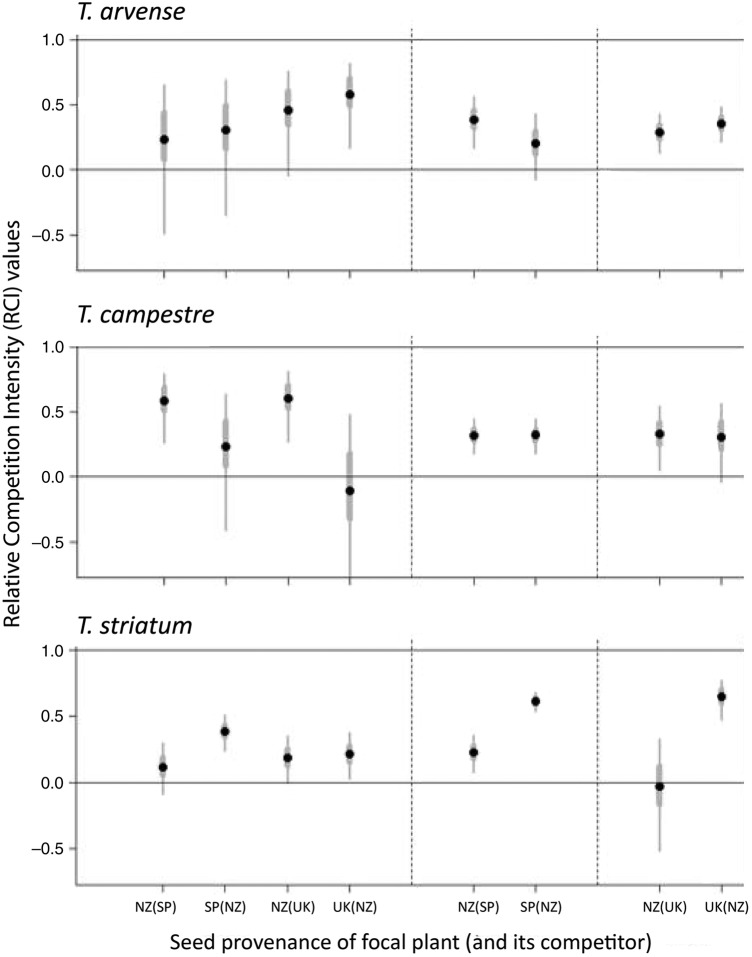
Relative competition intensity indices for plants from the introduced provenance (New Zealand, NZ) and the native-range provenances (Spain, SP, and the United Kingdom, UK) for three *Trifolium* species grown in pots inoculated with soil from each location. The RCI index is calculated as follows: RCI_A(B)_ = (GR_A_ − GR_A(B)_)/GR_A_, where GR_A_ is the growth rate of provenance A grown alone and GR_A(B)_ is the growth rate of provenance A grown in competition with provenance B. Higher RCI values (up to a maximum of 1) indicate a stronger competitive effect of provenance B on provenance A; zero indicates no effect of competition. Error bars are 50 % (thick grey bars) and 95 % (thin bars) confidence intervals.

**Figure 3.**
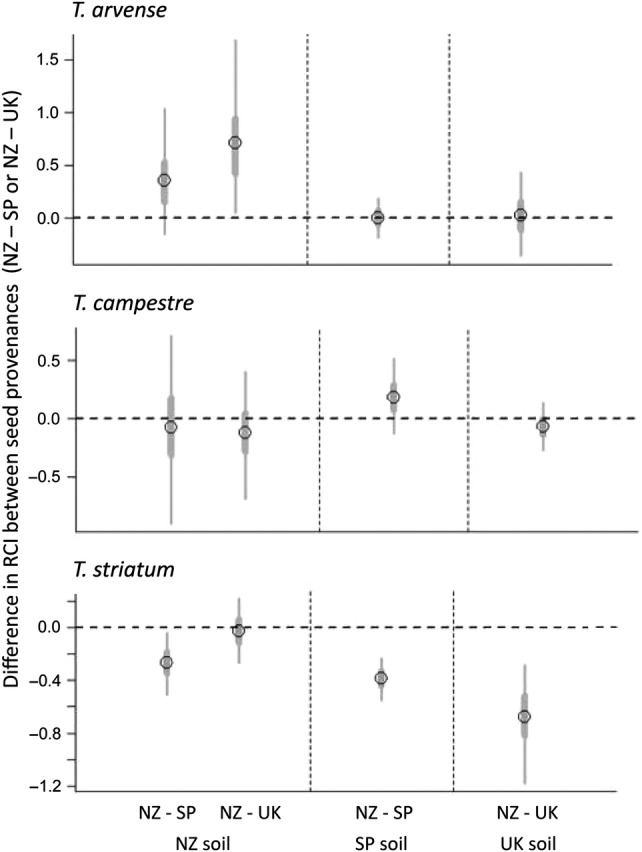
The difference in RCI values between plants from the introduced provenance (New Zealand, NZ) and each native-range provenance (Spain, SP, or the United Kingdom, UK) for three *Trifolium* species grown in pots inoculated with soil from each location. A value <0 indicates that the introduced provenance was more competitive than the native provenance. Error bars are 50 % (thick grey bars) and 95 % (thin bars) confidence intervals.

Competition significantly reduced plant growth rates, with plants in paired-plant pots growing more slowly than plants in single-plant pots by an average of 35 % (*F*_1,384_ = 49.13; *P* < 0.001), confirming that our paired-plant treatments had created competitive conditions. With a few exceptions, native and introduced provenances had similar competitive ability (Fig. 2). In New Zealand soils, New Zealand provenances of *T. striatum* were slightly more competitive than native conspecifics from Spain, consistent with EICA, but for the other two species, there was either no difference between provenances or, in the case of *T. arvense*, native UK plants were slightly more competitive than plants from the New Zealand (Fig. 3)—the opposite of the EICA prediction.

Among the native-range soils, the only significant difference in inter-provenance RCI values was that *T. striatum* plants from the introduced provenance were more competitive than those from the native range—also opposing the EICA prediction. Overall, the growth rate of provenances in the single-plant trials was not positively correlated with their competitive ability. Rather, there was a significant negative correlation between the magnitude of the differences in growth rate between provenances and the magnitude of the differences in RCI values between provenances (Pearson's correlation = −0.69; *P* = 0.01; *N* = 12). Thus, although the growth-rate differences between plants from native and introduced provenances were sometimes substantial, they did not correspond to differences in competitive ability.

## Discussion

We found no consistent evidence for increased competitive ability among three widely naturalized *Trifolium* species in New Zealand. Of the 12 comparisons of competitive ability between plants from native and introduced provenances, only one was in the direction predicted; the remainder either showed no difference in competitive ability between provenances (nine comparisons) or a significant difference in the direction opposite to that predicted (two comparisons). This result was unexpected given the substantially lower growth rates of two species from the introduced provenance when grown in soil from the native range. Although our ability to generalize our specific results is inherently limited by the small number of populations used in each range, our results do clearly show that the growth rates of plants from the introduced provenance in native soils do not necessarily translate to competitive ability, as was assumed in many previous tests of the EICA hypothesis (Felker-Quinn *et al.* 2013).

### Competition in the context of invasion

We suggest three explanations for the lack of increased competitive ability in this system. First, it is possible that these species do not experience a net effect of enemy release in line with the predictions of EICA. This may occur either because the antagonistic biota in the introduced range exert similar selective pressures on defence maintenance as the biota in the native range or because enemy pressure is lower but beneficial microbes (e.g. rhizobia and arbuscular mycorrhizal fungi) are also lacking and offset the benefit of enemy release. In New Zealand, naturalized *Trifolium* rhizobia are often poor nodulators (Hastings *et al*. 1966), whereas microbial pests are common in the rhizospheres of agricultural *Trifolium* (Skipp and Christensen 1983; Wratt and Smith 1983; Skipp and Watson 1987).

A second explanation for the lack of increased competitive ability is that *Trifolium*, which are globally widespread, may already be excellent competitors and are not under selective pressure to adapt mechanisms to improve resource acquisition in shared environments (Craine and Dybzinski 2013). In their native range, *Trifolium* typically co-occur with congeners (Gillett and Taylor 2001), and in New Zealand, 16 non-agricultural species of *Trifolium* have naturalized widely (Gravuer 2004) and typically co-occur with competitive forbs and grasses as well as perennial *Trifolium* (Boswell *et al.* 2003; Maxwell 2013). Adaptation for increased competitive ability should not be expected in every plant invasion scenario, particularly if the invaded environment is rich in resources (e.g. after disturbance) or competition is lower than in the invader's native range (Sun *et al.* 2014). Alternatively, in stressful or low-resource environments, species may evolve to grow when resources are available and remain viable when resources are scarce or competition is high (Grime *et al.* 2014), as appears to be the case for invasive *Hieracium* spp. (Asteraceae) in New Zealand hill country. These invaders grow on poor soils with highly competitive pasture species (including *T. repens*) yet do not appear to experience competitive effects (Scott and Sutherland 1993).

A final, potentially more parsimonious explanation is that the EICA hypothesis does not apply here and it cannot be considered a general explanation for the success of plant invaders. A recent review of the EICA literature by Felker-Quinn *et al.* (2013) revealed abundant evidence of adaptation among introduced plants, but found that support for EICA remains equivocal.

### Growth rate versus competitive ability

A key strength of our study is that we did not rely on growth rate as a proxy for competitive ability and instead directly measured the relative competitive ability of introduced and native provenances using intraspecific competition experiments. While previous EICA tests have assumed that higher growth rate equates to greater competitive ability in the invaded range (Blossey and Nötzold 1995; Franks *et al.* 2008; Handley *et al.* 2008), we found the opposite: species with a larger difference in growth rate between provenances when grown singly tended to have a smaller difference in relative competitive ability when grown in intraspecific pairings. Although our study only compared conspecifics, the lack of positive correlation between growth and competitive ability suggests that we need to be cautious in assuming that growth rate and plant size are always reliable surrogates for performance in competitive scenarios. Relatively few EICA competition studies have analysed both growth and competition; but of those that have, only a handful found correlations between increased growth and increased competitive ability (Vilà *et al.* 2003; Bossdorf *et al.* 2004; McKenney *et al.* 2007; Ridenour *et al.* 2008; Graebner *et al.* 2012; Oduor *et al.* 2013). Clearly, more direct measures of competition are needed to properly test for evidence of post-naturalization changes in competitive ability.

### Integrating soil microbiota

The differences we observed in RCI values for provenances grown in the presence of different rhizosphere microbial communities illustrate how soil biota can affect both growth and competitive ability. Most glasshouse tests of the EICA hypothesis use soils that are sterilized, commercially sourced or neutral (i.e. collected from a particular range, but not cultivated by conspecifics), despite clear evidence that plant performance is intimately tied to interactions with soil antagonists, mutualists and saprophytes (Wardle *et al.* 2004; Inderjit and van der Putten 2010; Inderjit and Cahill 2015). Such synergistic or interacting components must be incorporated into plant-competition study designs. The EICA hypothesis has mainly been developed from an aboveground perspective (Cipollini *et al.* 2005; Hull-Sanders *et al.* 2007; Doorduin and Vrieling 2011; Bekaert *et al.* 2012; Dawson 2015); it is now time to more fully integrate the role of soil microbial communities to better address the potential effects of these interactions on the post-naturalization performance and competitive ability of non-native plants.

## Conclusions

We investigated the growth rates and intraspecific competitiveness of three widespread non-native plants when grown with rhizosphere microbiota cultivated by conspecifics in soils from the native and introduced ranges. We found no evidence to support increased competitive ability and thus reject the EICA hypothesis in this system. Although our ability to generalize is limited because we included only one population of each species from each location, our study revealed an important finding: growth rate may not always be a reliable surrogate for competitive ability—specifically among conspecifics. We suggest that the use of (i) intraspecific pairings, (ii) direct tests of competition and (iii) the integration of soil microbial communities from each range will provide more powerful and informative tests of the EICA hypothesis.

## Sources of Funding

This work was funded by a grant from the Marsden Fund administered by the Royal Society of New Zealand and further supported by the Bio-Protection Research Centre, Lincoln, New Zealand.

## Contributions by the Authors

N.S., R.P.D., W.H.v.d.P. and P.E.H. designed the experiments; N.S., K.J.M. and C.W. conducted the field and glasshouse experiments; N.S. and R.P.D. performed the statistical analyses; all authors contributed to writing the manuscript.

## Conflict of Interest Statement

None declared.

## Supporting Information

The following additional information is available in the online version of this article —

**Figure S1.** The mean root nodulation scores of plants from each provenance in each soil. Scores are shown for informational purposes only; any differences in growth rate associated with nodulation by nitrogen-fixing symbionts were removed prior to analyses using the linear mixed-effects models.

**Table S1.** General information on the three study species.

**Table S2.** (A) The source locations for the rhizosphere soils and (B) the source locations for the seeds.

**Table S3.** The germination conditions for each species.

**Table S4.** The scoring system for nodulation with nitrogen-fixing symbionts.

**Table S5**. The R code for the linear mixed-effects models.

Additional Information
