# Supplementary material for: No difference in the competitive ability of introduced and native Trifolium provenances when grown with soil biota from their introduced and native ranges
Source: AoB Plants. 2016 Mar 11;8:plw016. doi: 10.1093/aobpla/plw016 (PMC4833883; doi:10.1093/aobpla/plw016)
Supplement: Additional Information [file supp_8_plw016_index.html]

No difference in the competitive ability of introduced and native Trifolium provenances when grown with soil biota from their introduced and native ranges — Additional Information 

# No difference in the competitive ability of introduced and native *Trifolium* provenances when grown with soil biota from their introduced and native ranges

## Additional Information

Additional Information

- Additional Information - Doc file
