# Supplementary material for: No difference in the competitive ability of introduced and native Trifolium provenances when grown with soil biota from their introduced and native ranges
Source: AoB Plants. 2016 Mar 11;8:plw016. doi: 10.1093/aobpla/plw016 (PMC4833883; doi:10.1093/aobpla/plw016)
Supplement: Additional Information [file supp_plw016_plw016supp.doc]

**Supporting Information**

No difference in the competitive ability of introduced and native *Trifolium* provenances when grown with soil biota from their introduced and native ranges


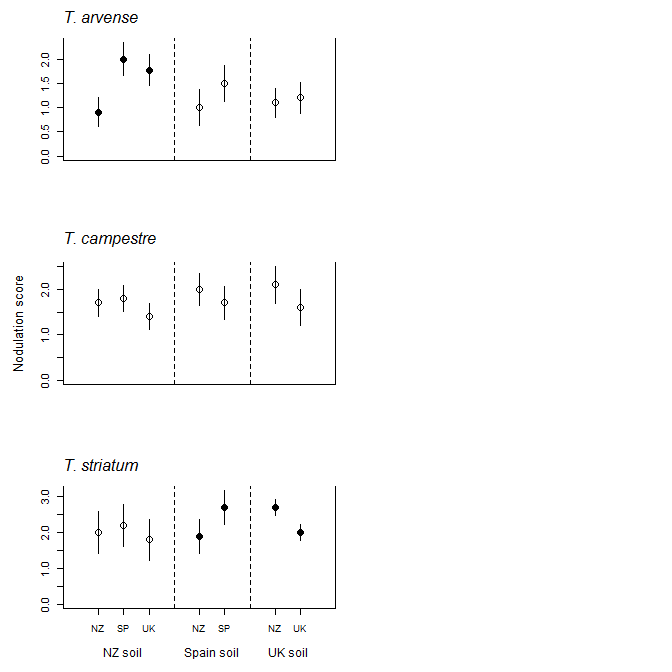


Figure S1. Nodulation scores for *Trifolium* plants from introduced (New Zealand, NZ) and native Spain (SP) and native UK (UK) seed provenances in the competition paired-pot treatments. Filled circles represent significant (*P = <* 0.05) differences between the non-native and native provenances. Differences in growth related to nodulation with nitrogen-fixing symbionts were controlled statistically in the linear mixed-effects models.

Table S1. Summary information for the three species of *Trifolium* used in this study.

|  | |  |  | Distribution | |  | Performance in glasshouse trials | | | |
| --- | --- | --- | --- | --- | --- | --- | --- | --- | --- | --- |
|  | |  |  |  | |  | | |  |  |
| **Species** | **Years naturalised in New Zealand*** | | **Date  naturalised in  New Zealand*** | **Non-native range** ǂ* | **Native  range** ¶* | **Nodule score  (Mean ± S.E.)** | | **Dry-weight biomass  (Mean g  ± S.E.)** | **Seed size  (Mean mg  ± S.E.)** | **Seed size  (Mean mg  ± S.E.)** |
|  |  | |  |  |  |  | |  |  |  |
| *T. arvense* | 138 | | 1876 | 83 | 26.6 | 1.6 ± 0.1 | | 0.6 ± 0.1 | 0.38 ± 0.01 | 0.38 ± 0.01 |
| *T. campestre* | 147 | | 1867 | 46 | 20.1 | 1.8 ± 0.1 | | 0.8 ± 0.1 | 0.30 ± 0.00 | 0.30 ± 0.00 |
| *T. striatum* | 138 | | 1876 | 44 | 10.1 | 1.6 ± 0.1 | | 2.2 ± 0.1 | 1.83 ± 0.07 | 1.83 ± 0.07 |

ǂ Number of 10 x 10 km NZMS260 grids occupied by at least one population; Gravuer 2004
¶ Area estimate (x 10^12^ km^2^); Gravuer 2004
* Data from Gravuer 2004

Table S2A. Field site location details for the rhizosphere soil used to inoculate the glasshouse pots. Local abundance was estimated using a modified DAFOR (Dominant, Abundant, Frequent, Occasional, Rare) scale where 0 = occasional, 1 = frequent, 2 = abundant.

| **Species** | **Country** | **Location** | **Latitude** | **Longitude** | **Local abundance** | **Mean pH** |
| --- | --- | --- | --- | --- | --- | --- |
|  |  |  |  |  |  |  |
| *Trifolium arvense* | Spain | Parador de Oriel, Aragon | 42.52767 | -00.53161 | 1 | 6.12 |
|  | Spain | Benabarre, Huerrios Mtns, Aragon | 42.13871 | 00.47665 | 2 | 6.49 |
|  | Spain | Benabarre, Huerrios Mtns, Aragon | 42.16830 | 00.45163 | 2 | 5.61 |
|  | Spain | Blanes, Catalonia | 41.66857 | 02.76699 | 1 | 7.77 |
|  | Spain | El Port de la Selva, Catalonia | 42.32964 | 03.19938 | 2 | 7.68 |
|  | UK | Bournemouth, Monkey Island | 50.71949 | -01.85750 | 1 | 5.36 |
|  | UK | Gosport, Browndown | 50.79236 | -01.19320 | 2 | 7.14 |
|  | UK | Devon, near Torquay | 50.45835 | -03.49138 | 0 | 4.81 |
|  | UK | Gower, Pennard Burrows | 51.57602 | -04.09137 | 0 | 5.24 |
|  | UK | Kenfig | 51.51550 | -03.72777 | 1 | 6.47 |
|  | NZ | Christchurch, Canterbury | -43.53654 | 172.60981 | 1 | 5.54 |
|  | NZ | Kaitorete Spit, Banks Pen. | -43.82550 | 172.69896 | 1 | 5.31 |
|  | NZ | Birdlings Flat, Banks Pen. | -43.81554 | 172.69999 | 2 | 5.26 |
|  | NZ | New Brighton, Canterbury | -43.52554 | 172.72266 | 1 | 6.00 |
|  | NZ | South Brighton, Canterbury | -43.51955 | 172.71967 | 0 | 7.03 |
|  |  |  |  |  |  |  |
| *Trifolium campestre* | Spain | Zubillaga, Alaba, Basque Country | 42.71492 | -02.9784 | 2 | 7.70 |
|  | Spain | Pancorbo, Castilla y León | 42.63908 | -03.10591 | 2 | 7.52 |
|  | Spain | Barcina del Barco, Castilla Y León | 42.78152 | -03.22899 | 2 | 7.62 |
|  | Spain | Rio Ebro, Castilla y León | 42.76448 | -03.18876 | 2 | 7.95 |
|  | Spain | Embalse de Sobrón, Basque Country | 42.76807 | -03.10081 | 2 | 7.60 |
|  | UK | Gosport, Browndown | 50.79231 | -01.19333 | 2 | 7.34 |
|  | UK | Gosport, Browndown | 50.79346 | -01.19466 | 2 | 7.25 |
|  | UK | Swansea, Crymlyn | 51.62422 | -03.84303 | 0 | 6.75 |
|  | UK | Devon, near Chudleigh | 50.61418 | -03.62361 | 0 | 6.58 |
|  | UK | Kenfig, near Sharkham Point | 51.50608 | -03.74308 | 2 | 7.26 |
|  | NZ | Christchurch, Canterbury | -43.53654 | 172.60981 | 0 | 6.68 |
|  | NZ | Chorlton Road, Banks Pen. | -43.67533 | 173.04443 | 0 | 5.72 |
|  | NZ | Western Valley Road, Banks Pen. | -43.74696 | 172.79556 | 1 | 5.71 |
|  | NZ | Streeters Road | -43.73616 | 172.62569 | 1 | 4.73 |
|  | NZ | Big Hill Road, Banks Pen. | -43.70170 | 173.06485 | 2 | 5.79 |
| *Trifolium striatum* | Spain | Parador de Oriel, Aragon | 42.52767 | -00.53161 | 0 | 6.08 |
|  | Spain | Benabarre, Huerrios Mtns, Aragon | 42.13871 | 00.47665 | 1 | 5.96 |
|  | Spain | Sant Pere de Rodes, Catalonia | 42.32933 | 03.15826 | 0 | 5.64 |
|  | Spain | Girona, Catalonia | 42.29192 | 03.15193 | 1 | 6.85 |
|  | Spain | Girona, Catalonia | 42.36454 | 03.02926 | 2 | 5.82 |
|  | UK | Bournemouth, Monkey Island | 50.71936 | -01.85817 | 1 | 5.14 |
|  | UK | Gosport | 50.79219 | -01.18068 | 0 | 6.96 |
|  | UK | Devon, south of Berryhead | 50.38211 | -03.49983 | 0 | 6.85 |
|  | UK | Gower, Broadpool | 51.59786 | -04.15189 | 2 | 5.17 |
|  | UK | Gower, Pennard Burrows | 51.57591 | -04.09119 | 0 | 5.05 |
|  | NZ | Birdlings Flat, Banks Pen. | -43.81554 | 172.69999 | 1 | 5.24 |
|  | NZ | Heathcote Quarry Track, Banks Pen. | -43.57186 | 172.71675 | 1 | 5.41 |
|  | NZ | Governors Bay, Banks Peninsula | -43.63209 | 172.65205 | 2 | 5.27 |
|  | NZ | Ataahua Domain, Canterbury | -43.77608 | 172.64566 | 0 | 4.44 |
|  | NZ | Big Hill Road, Banks Pen. | -43.70170 | 173.06485 | 2 | 5.89 |

Table S2B. Seed source locations for the three species of *Trifolium* used in this study.

| **Species** | **Country** | **Region** | **Source** | **Latitude** | **Longitude** | **Collected** |
| --- | --- | --- | --- | --- | --- | --- |
|  |  |  |  |  |  |  |
| *T. arvense* | UK | NA | Herbiseed | NA | NA | NA |
|  | Spain | Blanes, Catalonia | Field | 41.66857 | 02.76699 | June 2012 |
|  | New Zealand | Kaitorete Spit, Canterbury | Field | -43.82550 | 172.69896 | Feb .2012 |
|  |  |  |  |  |  |  |
| *T. campestre* | UK | Crymlyn Burrows, Wales | Field | 51.62422 | -03.84303 | June 2012 |
|  | Spain | Gorliz Beach, Basque Country | Field | 43.41476 | -02.94018 | June 2012 |
|  | New Zealand | Banks Peninsula, Canterbury | Field | -43.70170 | 173.06485 | Feb. 2012 |
|  |  |  |  |  |  |  |
|  |  |  |  |  |  |  |
| *T. striatum* | UK | Bournemouth, England | Field | 50.719356 | -01.85740 | June 2012 |
|  | Spain | Huerrios Mtns, Aragon | Field | 42.14666 | -00.47159 | June 2012 |
|  | New Zealand | Banks Peninsula, Canterbury | Field | -43.63209 | 172.65205 | Feb. 2012 |

Table S3. Germination conditions used in the light- and temperature-controlled cabinets for the three species of *Trifolium* in this study.

| **Species** | **Days in paper at 4 °C** | **Light: Dark** | **Temperature range** | **Weeks to transplant** |
| --- | --- | --- | --- | --- |
| *T. arvense* | 2 | 8:16 | 16-18 °C | 4 |
| *T. campestre* | 4 | 8:16 | 16-18 °C | 4 |
| *T. striatum* | 0 | 8:16 | 16-18 °C | 3 |
|  |  |  |  |  |

*Table S4. Scoring index used for categorizing root nodulation of nitrogen-fixing symbionts on all species of Trifolium. Based loosely on Corbin et al. 1977.*

|  | **Description** | **Level of functionality** |
| --- | --- | --- |
|  |  |  |
| 0_a_ | No nodules | None |
|  |  |  |
| 0_b_ | Nodules present but all < 1 mm wide and/or  lacking pigment, suggesting parasitism | Potentially negative (parasitic) |
|  |  |  |
| 1 | Few nodules, only at distal portions of root system;  mostly < 1 mm wide, light pink | Low |
|  |  |  |
| 2 | Nodules scattered throughout root system;  many > 1 mm wide, light pink to red | Medium |
|  |  |  |
| 3 | Abundant nodules, particularly in the top 2 mm  of root crown; many > 1 mm wide, red to purple indicating presence of the oxygen-carrier leghaemoglobin. | High |

Table S5. R code for linear mixed-effect (lme) models used to compare (A) single plant performance (growth rate) and (B) competitive ability (RCI value) of plants from native and non-native seed provenances. Models were fit with the lmer function of the R package “arm” (Gelman & Su 2014), run separately for each soil. (A) Models to analyse differences in growth between native and non-native plants in non-competitive conditions (single plant treatments) single-plant was run with (m1-m3) and without (m1a-m3a) the fixed factor “seed” and the pair of models was analysed via ANOVA. (B) Competition models were used to extract the mean growth rate and associated uncertainty for each seed provenance and seed-provenance combination having accounted for site effects. Mean growth rates and their uncertainties were used to calculate RCI indices via a simulation approach. (C) Model variables used in the linear mixed-effects models.

**Model R code**

**(A) Growth analysis (singly-grown plants only)**

Log-transformed growth rate as a factor of seed provenance in NZ soil m1 <- lmer(log(biom) ~ seed + (1|site)+nods2, data=as, subset=soil=="NZ"
*m1 with “seed” removed m1a <- lmer(log(biom) ~ 1 + (1|site)+nods2, data=as, subset=soil=="NZ"

Log-transformed growth rate as a factor of seed provenance in Spanish soil m2 <- lmer(log(biom) ~ seed + (1|site)+nods2, data=as, subset=soil=="SP"
*m2 with “seed” removed m2a <- lmer(log(biom) ~ 1 + (1|site)+nods2, data=as, subset=soil=="SP"

Log-transformed growth rate as a factor of seed provenance in UK soil m3 <- lmer(log(biom) ~ seed + (1|site)+nods2, data=as, subset=soil=="UK"
*m3 with “seed” removed m3a <- lmer(log(biom) ~ 1 + (1|site)+nods2, data=as, subset=soil=="UK"

**(B) Competition analysis (single vs paired growth)**

Log-transformed growth rate as a factor of group in NZ soil m4<- lmer(log(biom) ~ group-1 + (1|site), data=as, subset=soil=="NZ")
Log-transformed growth rate as a factor of group in Spanish soil m5<- lmer(log(biom) ~ group-1 + (1|site), data=as, subset=soil=="SP")
Log-transformed growth rate as a factor of group in UK soil m6<- lmer(log(biom) ~ group-1 + (1|site), data=as, subset=soil=="UK")

**(C) Model variable Purpose**

seed A fixed factor to test for differences in performance
between seed provenances

site A random factor to control for differences between
the five soil replicates in each soil (NZ, Spain, UK)

nods2 A fixed factor to control for the effect of colonisation
by nitrogen-fixing symbionts on growth rate

group A variable grouping plant origin (NZ, Spain, UK)
pot treatment (single vs. paired) and the origin
of the competitor plant (NZ, Spain, UK)
